# The prognostic value of fat invasion and tumor expansion in the hilar veins in pT3a renal cell carcinoma

**DOI:** 10.1007/s00345-021-03638-0

**Published:** 2021-02-27

**Authors:** Viktoria Stühler, Steffen Rausch, Katharina Kroll, Marcus Scharpf, Arnulf Stenzl, Jens Bedke

**Affiliations:** 1 Department of Urology, University of Tuebingen, Tübingen, Germany; 2 Department of Pathology, University of Tuebingen, Tübingen, Germany

**Keywords:** Fat invasion, Hilar vein involvement, pT3a stage, Renal cell carcinoma

## Abstract

**Purpose:**

The 7th TNM classification summarizes renal cell carcinoma (RCC) with perirenal (PFI) and/or sinus fat invasion (SFI) as well as hilar vein involvement (RVI) as pT3a tumors. In this study, we aimed to determine the prognostic value of fat invasion (FI) in the different compartments and RVI for medium-term cancer-specific-survival (CSS) in pT3a RCC.

**Materials and methods:**

Patients with pT3a RCC were identified using an institutional database. All original pathological reports were reclassified according to the 7th TNM edition. The prognostic value of FI as well as divided into PFI, SFI, combined PFI + SFI, and RVI for CSS was assessed using univariate and multivariate Cox-regression analysis. Survival was estimated using the Kaplan–Meier method.

**Results:**

Median follow-up in 184 pT3a tumors was 38 months. FI was detectable in 153 patients (32.7% PFI, 45.1% SFI, 22.2% PFI + SFI), 31 patients showed RVI alone. Combined PFI + SFI increased the risk of cancer-related death compared to PFI (HR 3.11, *p* < 0.01), SFI (HR 1.84, *p* = 0.023) or sole RVI (HR 2.12, *p* = 0.025). In multivariate analysis, a combined PFI + SFI vs. PFI or SFI as the only compartment involved was confirmed as independent prognostic factor (HR 1.83, *p* = 0.029). Patients with FI and simultaneous RVI had significantly shorter CSS (HR 2.63, *p* < 0.01). In an unweighted model, the difference between patients with combined PFI + SFI and RVI and those with PFI alone was highest (HR 4.01, *p* = 0.029).

**Conclusions:**

These results underline the subdivision of pT3a RCC depending on the location of FI and RVI for patient stratification.

## Introduction

**Fig. 1:**
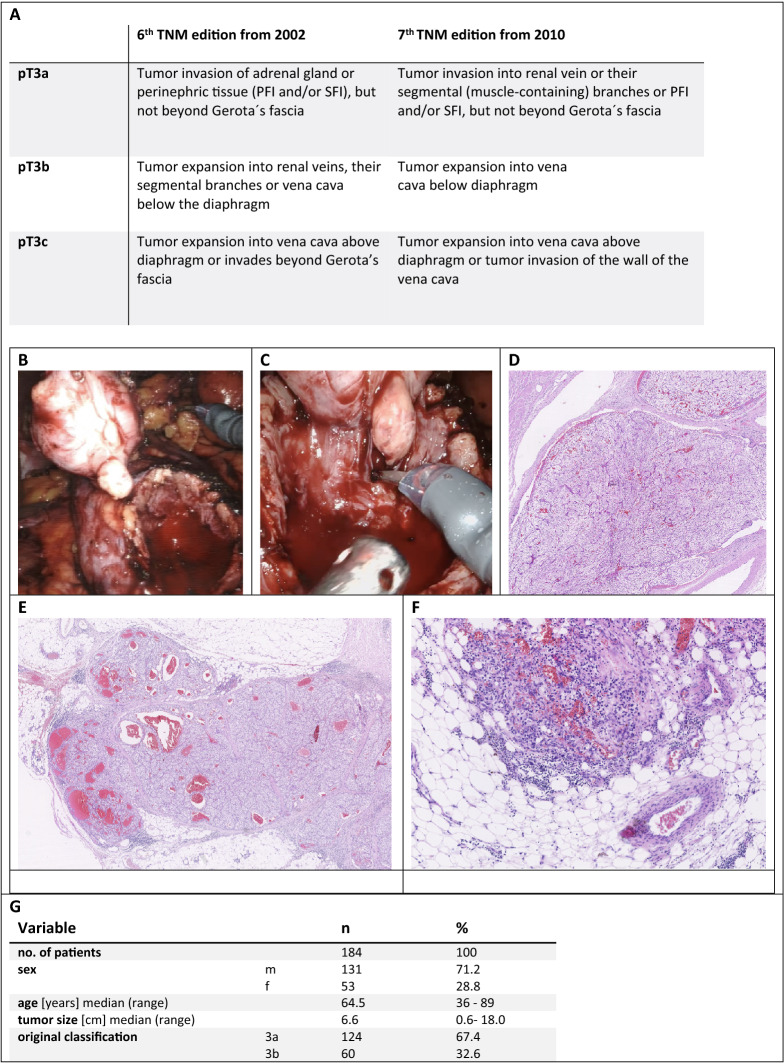

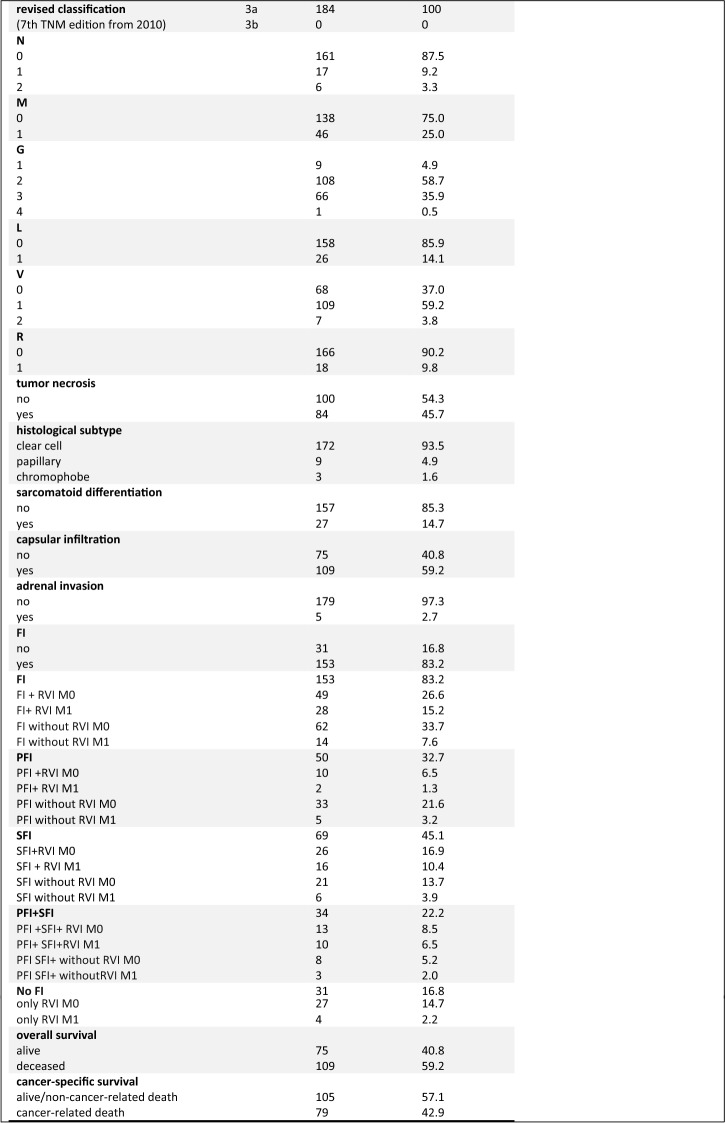
**a** CSS of the total collective (median 94 months). **b** CSS of patients with FI depending on RVI, *p* < 0.001. **c** CSS depending on affected area of FI in the total population; SFI vs. PFI: *p* = 0.079, PFI vs. PFI + SFI: *p* < 0.001, SFI vs. PFI + SFI: *p* = 0.023; no FI only RVI vs. PFI + SFI: *p* = 0.025. **d** CSS depending on affected area of FI in the M0 subcollective; SFI vs. PFI: *p* = 0.667, PFI vs. PFI + SFI: *p* = 0.226, SFI vs. PFI + SFI: *p* = 0.408; no FI only RVI vs. PFI + SFI: *p* = 0.559. All *p*values for the overall comparison (log-rank test); significance in pairwise comparison. **e** Unweighted prognostic risk stratification model 1: CSS of patients depending on the variables M-stage, L-stage, and RVI; M0 L1 RVI1 vs. M0 L0 RVI0: *p* < 0.001. **f** Unweighted prognostic risk stratification model 2: CSS of patients depending on the variable M-stage, PFI, SFI, and combined PFI and SFI with or without RVI; PFI + RVI M0 vs. PFI without RVI M0: *p* = 0.043; SFI + RVI M0 vs. SFI without RVI M0: *p* = 0.409; PFI + SFI + RVI M0 vs. PFI + SFI without RVI M0: *p* = 0.202. **g **Tabular summary of the models 1 and 2. **h** Flowchart showing the process of patient selection for this retrospective study, starting from the total cohort of patients after radical or partial nephrectomy for RCC between 1993 and 2003 at the Department of Urology, University of Tuebingen. *FI* fat invasion, *L* invasion into lymph vessels, *M* distant metastasis, *PFI* perirenal fat invasion, *RVI* renal vein involvement, *SFI* hilar fat invasion

Overall survival of patients with renal cell carcinoma (RCC) varies widely. The likelihood for patients with localized RCC treated in curative intention with tumor resection to suffer relapse with lymph node or distant metastases is up to 30% [1]. After tumor resection, an accurate assessment of the risk of relapse is important to offer a risk-adapted follow-up frequency and, if available, the possibility of adjuvant therapy, taking into account the outstanding study results with immune checkpoint inhibitors. Tumor invasion in the renal fat is often divided into perirenal (PFI) and perihilar (SFI) fat invasion and is detected in 5.1–18.5% of cases [2, 3]. The 2010 TNM classification led to a major change in RCC classification. Up to this time, tumors with invasion of the perinephric tissue or the adrenal gland were classified as pT3a tumors, and those with expansion into the hilar veins, their segmental branches or the vena cava below the diaphragm as pT3b tumors. Due to the revision of the TNM classification in 2010, tumors with FI and even extension into the hilar veins or their segmental (muscle-containing) branches are now summarized as pT3a tumors. In the new classification, a pT3b tumor is characterized by a tumor expansion into the vena cava below the diaphragm [4]. The changes in TNM classification are summarized in Fig. 1 and illustrated with exemplary histologic images. However, the prognostic value of FI with subdivision in the different fat compartments PFI and SFI with or without hilar vein involvement (RVI) is controversially discussed.

In this study, we examined the prognostic value of FI, divided in PFI, SFI, and combined PFI + SFI, and RVI for medium-term cancer-specific survival (CSS) in patients with pT3a RCC to improve risk stratification. These results shall help to adjust the follow-up frequency after kidney surgery, and to identify patients at high risk of recurrence who may benefit from adjuvant treatment strategies.

## Patients and methods

**Fig. 2:**
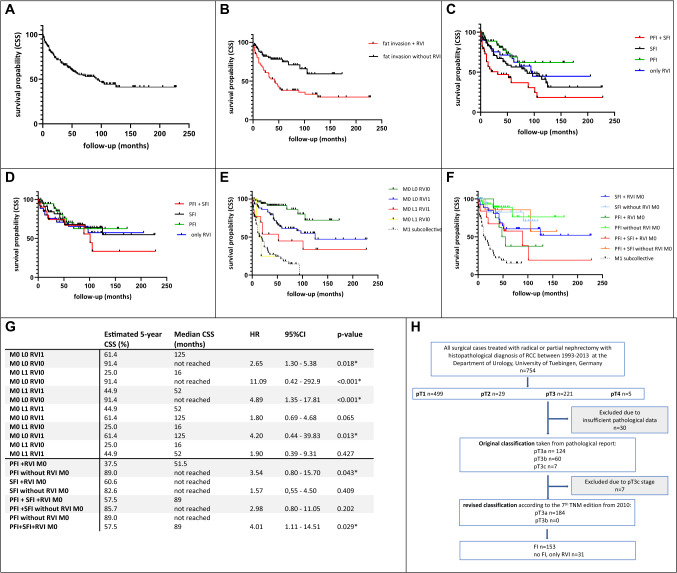
**a** 6th TNM edition from 2002 and 7th TNM edition from 2010 for pT3 subclassification of RCC. Below, two exemplary intraoperative images of RCC with invasion into the segmental (muscle-containing) branches of the hilar veins are shown in the top row (**b**, **c**) with the matching histologic image (**d**). Below that, a histologic overview image of an RCC with perirenal fat invasion is shown with corresponding magnification on the right side (**e**, **f**). **g** Detailed patient characteristics of the investigated pT3a RCC cohort. *FI* fat invasion, *G* grading, *L* invasion into lymph vessels, *M* distant metastasis, *N* regional lymph nodes, *PFI* perirenal fat invasion, *R* resection status, *RVI* renal vein involvement, *SFI* hilar fat invasion, *T* primary tumor, *V* invasion into veins

A flowchart showing the process of patient selection for this retrospective study from a total cohort with 754 patients after radical or partial nephrectomy for RCC between 1993 and 2003 at the Department of Urology, University of Tuebingen, Germany, is deposited in Fig. 2. Primary histopathologic analysis was carried out by uro-pathologists, whereas re-assessment according to 7th TNM edition from 2010 was performed by the review of original pathological reports by two authors (V.S. and J.B.). In case of discrepancy, reports were re-evaluated by the uro-pathologist (M.S.). Finally, 184 patients with histological diagnosed pT3a RCC were included in the study. Data were collected in an institutional database and included documentation of sex, age, height, weight, nicotine or alcohol use, time of death or time of last follow-up, and cause of death (RCC-related or RCC-independent). The following tumor-specific parameters were also collected: fat invasion (PFI, SFI, or combined PFI + SFI), histological subtype, tumor size, TNM classification according to UICC 2010, grading, L-, V-, and R-stages, the presence of necrosis and/or sarcomatoid parts and capsular or adrenal invasion. Because fat invasion (FI) correlated with N- and M-stages in preliminary analyses (data not shown), cM1 patients were also included in first analyses. The last follow-up was carried out in January 2020. The study was approved by the local ethics committee (078/2012/B02).

The primary aim of this study was the retrospective evaluation of the prognostic value of FI into the different renal compartments in correlation with or without RVI for medium-term CSS in pT3a RCC. All patients underwent surgical resection of the tumors renal mass according to institional surgical guidelines, in which nephron sparing surgery is recommend compared to radical nephrectomy if technical feasible either by open or laparoscopic approach.

### Statistical analysis

In a first step, we performed a descriptive statistic of the clinicopathological parameters. The prognostic influence of FI as well as divided into the different fat compartments or a sole RVI on CSS was investigated using univariate and multivariate Cox-regression analysis. Factors that showed significance in univariate analysis were evaluated using multivariate models. Survival analyses were estimated using the Kaplan–Meier method and significant differences were determined using the log-rank test for univariate analysis. All statistical analyses were performed with SPSS version 26 (released 2019, Armonk, NY: IBM Corp) and Windows Microsoft Excel (Office 12). Statistical significance was defined as *p* < 0.05.

## Results

This study involved 184 consecutive pT3a RCC patients. Due to the re-classification according to the 7th TNM edition from 2010, 60 originally classified pT3b tumors were reclassified as pT3a tumors. Patient characteristics are given in Fig. 1. The results of univariate analysis of clinicopathological parameters showing significant differences in CSS, such as tumor diameter, grading, L-, V-, and R-stages, tumor necrosis, sarcomatoid differentiation, capsular infiltration, N- and M-stages are listed in Table 1A. Because of a strong imbalance (*n* = 172 for clear cell RCC and *n* = 12 for non-clear cell RCC), histological subtype was not included in the univariate and multivariate Cox-regression models. Kaplan–Meier analyses of the 184 patients are shown in Fig. 2 and for the univariate Cox-regression analyses in Table 1. Classified according to the location of FI, PFI was present in 50 (32.7%), SFI in 69 (45.1%) and a combined PFI + SFI in 34 (22.2%) patients, respectively. 31 (16.8%) patients showed no FI, however, were classified as pT3a tumors due to RVI. Median follow-up was 32 months (0–228 months) with 85.3% follow-up rate in January 2020. During the follow-up period, 109 (59.2%) patients died, with 79 (42.9%) patients of cancer-specific death.

**Table 1:** **A** Estimated 2- and 5-year CSS, median CSS and univariate Cox-regression analysis of clinicopathological parameters, **B** univariate Cox-regression analysis of the different fat compartements, **C** model 1 (CSS)—multivariate analyses of clinical and pathological parameters with regard to FI, *n* = 18, **D:** model 2 (CSS, SFI vs. PFI), model 3 (CSS, PFI + SFI vs. PFI), model 4 (CSS, PFI + SFI vs. SFI), and model 5 (CSS, combined PFI + SFI vs. PFI or SFI as sole involved compartment)—multivariate analyses of clinical and pathological factors of the total collective

A						
	Estimated 2-year CSS (%)	Estimated 5-year CSS (%)	Median CSS (months)	Univariate Cox-regression analysis		
				HR	95% CI	*p* value
*Whole collective*	71.2	55.8	94.00			
FI	70.9	53.5	91.0			
No FI, only RVI	75.3	66.9	97.0	1.18	0.67–2.07	0.591
FI with RVI	61.0	38.1	42.0			
FI without RVI	82.1	75.0	Not reached	2.63	1.62–4.26	< 0.001*
*M0 subcollective*						
FI	86.1	70.0	Not reached			
No FI, only RVI	75.7	65.5	Not reached	0.87	0.40–1.87	0.397
*M1 subcollective*						
FI	33.0	15.1	14			
No FI, only RVI	75.0	50.0	67.5	1.99	0.88–4.49	0.139
PFI	88.8	70.00	Not reached			
SFI	70.9	56.5	87.0	For more details see Table 1B		
PFI + SFI	50.6	36.8	32.0			
*Tumor diameter*						
< Median	79.8	67.0	Not reached			
≥ Median	62.5	43.4	51	0.45	0.29–0.71	< 0.001*
*N*						
N0	76.0	61.5	125			
N1/N2	43.5	21.7	17	0.30	0.14–0.64	< 0.001*
*M*						
M0	84.0	68.3	Not reached			
M1	36.8	21.3	19	0.22	0.12–0.40	< 0.001*
*Grading*						
G1/G2	81.1	65.6	Not reached			
G3/G4	55.4	38.8	35	0.43	0.26–0.69	< 0.001*
*V*						
V0	83.3	77.7	Not reached			
V1/V2	65.3	45.6	51	0.36	0.23–0.56	< 0.001*
*L*						
L0	78.3	61.2	105			
L1	34.6	25.2	15.5	0.34	0.17–0.70	< 0.001*
*Tumor necrosis*						
No	83.6	63.8	125			
Yes	56.9	45.7	44	0.48	0.30–0.75	< 0.001*
*Sarcomatoid differentiation*						
No	75.0	60.0	125			
Yes	53.0	31.9	31	0.42	0.21–0.86	< 0.001*
*Capsular infiltration*						
No	81.4	61.7	125			
Yes	64.7	51.5	62	0.64	0.41–0.99	0.050
*R*						
0	74.3	58.5	97			
1/2	47.1	32.3	20	0.53	0.24–1.20	0.047*

B			
Patient population	HR	95% CI	*p* value
*Total collective*			
SFI vs. PFI	1.76	0.98–3.26	0.079
PFI + SFI vs. PFI	3.11	1.55–6.24	< 0.001*
PFI + SFI vs. SFI	1.84	1.01–3.35	0.023*
PFI + SFI vs. no FI, only RVI	2.12	1.09–4.13	0.025*
*M0 subcollective*			
SFI vs. PFI	1.20	0.53–2.69	0.667
PFI + SFI vs. PFI	1.75	0.67–4.57	0.226
PFI + SFI vs. SFI	1.57	0.64–3.83	0.408
PFI + SFI vs. no FI, only RVI	1.31	0.52–3.34	0.559
*M1 subcollective*			
SFI vs. PFI	2.47	0.95–6.41	0.125
PFI + SFI vs. PFI	3.66	1.37–9.77	0.019*
PFI + SFI vs. SFI	1.95	0.88–4.32	0.055
PFI + SFI vs. no FI, only RVI	3.48	1.35–9.02	0.004*

C			
	HR	95% CI	*p* value
FI	1.24	0.64–2.38	0.523
RVI	2.20	1.25–3.87	0.006*
M-stage	4.20	2.55–6.94	< 0.001*
L-stage	2.38	1.34–4.23	0.003*
Grading	1.10	0.62–1.93	0.744
Tumor necrosis	1.39	0.85–2.27	0.189
Sarcomatoid differentiation	1.57	0.84–2.95	0.161
Tumor diameter	1.36	0.84–2.21	0.213

D								
	Model 2 (CSS) SFI vs. PFI		Model 3 (CSS) PFI + SFI vs. PFI		Model 4 (CSS) PFI + SFI vs. SFI		Model 5 (CSS) PFI + SFI vs. PFI or SFI as sole involved compartment	
	HR (95% CI)	*p* value	HR (95% CI)	*p* value	HR (95% CI)	*p* value	HR (95%CI)	*p* value
PFI								
SFI	1.30 (0.58–2.91)	0.524						
PFI + SFI			1.75 (0.78–3.90)	0.172	1.75 (0.97–3.16)	0.062	1.83 (1.06–3.14)	0.029*
RVI	1.94 (0.93–4.06)	0.077	3.22 (1.31–7.90)	0.011*	1.52 (0.76–3.04)	0.240	2.23 (1.25–3.96)	0.007*
M-stage	3.76 (1.88–7.53)	< 0.001*	7.13 (2.77–18.35	< 0.001*	6.44 (3.17–13.09)	< 0.001*	5.28 (2.92–9.55)	< 0.001*
L-stage	2.13 (0.96–4.74)	0.065	3.68 (1.54–8.79)	0.003*	2.52 (1.29–4.93)	0.007*	2.63 (1.44–4.80)	0.002*
Grading	1.52 (0.64–3.66)	0.345	1.75 (0.78–3.91)	0.173	1.54 (0.77–3.07)	0.221	1.39 (0.76–2.57)	0.286
Tumor necrosis	0.83 (0. 41–1.66)	0.597	1.14 (0.48–2.70)	0.761	1.60(0.88–2.91)	0.124	1.22 (0.72–2.08)	0.460
Sarcomatoid differentiation	1.17 (0.45–3.01)	0.747	1.41 (0.54–3.69)	0.483	1.02 (0.44–2.35)	0.958	1.29 (0.64–2.57)	0.480
Tumor diameter	1.23 (0.65–2.33)	0.519	0.97 (0.43–2.20)	0.944	1.11 (0.61–2.02)	0.725	1.10 (0.64–1.88)	0.728

*FI* fat invasion, *G* grading, *L* invasion into lymph vessels, *M* distant metastasis, *N* regional lymph nodes, *PFI* perirenal fat invasion, *R* resection status, *RVI* renal vein involvement, *SFI* hilar fat invasion, *V* invasion into veins

*Statistically significant (*p* < 0.05)

The estimated 2- and 5-year CSS rates of the whole collective depending on FI and divided in the different fat compartments are summarized in Table 1A. For the whole collective, FI itself was not a significant parameter for CSS compared to patients with only RVI (HR 1.18, *p* = 0.591, see Table 1A). Patients with FI and simultaneous RVI had a significantly shorter CSS compared to patients with sole FI (median CSS 42 months vs. not reached, HR 2.63, *p* < 0.01). Further, there was a significant difference between the subgroup of patients with combined PFI + SFI and patients with no FI but RVI (32 vs. 97 months, HR 2.12, *p* = 0.025). The difference in CSS was not significant between patients with SFI or PFI (*p* = 0.079). In univariate analysis, a combined PFI + SFI significantly increased the risk of cancer-related death compared to PFI (HR 3.11, *p* < 0.01) or SFI (HR 1.84, *p* = 0.023), see Table 1B. The 2- and 5-year CSS rates for the subgroup of patients without distant metastases (M0, *n* = 138) as well as the univariate analysis of the various fat compartments showed no significant difference in CSS and are summarized in Table 1A, B. Data of the M1 subcollective (*n* = 46) should be interpreted with caution due to the small number of patients and are also given in Table 1B.

In the multivariate analyses, the parameters M-stage, tumor diameter stratified by the median, tumor grading (≥ 3/4), L-stage, tumor necrosis, sarcomatoid differentiation and RVI were adjusted in addition to FI and substratified in the various fat compartments. As shown in model 1 in Table 1C, FI was no independent prognostic factor for CSS (HR 1.24, *p* = 0.523). On the other hand, the values of M- and L-stages and RVI were identified as independent prognostic parameters for CSS. When considering the affected FI compartments in multivariate analyses (model 2–5 in Table 1D), combined PFI + SFI compared to PFI or SFI as single affected compartment was an independent prognostic factor for poorer tumor-dependent survival (HR 1.83, *p* = 0.029, see model 5). However, models 3 and 4 in Table 1D show that the presence of a combined PFI + SFI compared to an isolated PFI (HR 1.75, *p* = 0.172) or SFI (HR 1.75, *p* = 0.062) could not be identified as independent prognostic factors for CSS. However, RVI was identified as an independent prognostic factor for CSS in models 1, 3, and 5. Further, the presence of distant metastases and L-stage were identified as independent predictive factors for CSS in all multivariate Cox-regression models.

Finally, we created an unweighted prognostic risk stratification model for Kaplan–Meier analysis to further investigate the independent prognostic parameters M-stage, L-stage, and RVI, see Fig. 2e, g. This model stratified pT3a M0 patients into groups that differ significantly in their CSS. The subgroup of patients with FI M0 L0 RVI0 showed a significantly improved CSS compared to those with FI M0 L1 RVI1 (5-year CSS rate 91.4% vs. 44.9%, HR 4.89, *p* < 0.01). In our study, patients of the group M0 L1 RVI0 showed an even worse median CSS of 16 months. However, due to the small number of patients in this subgroup (*n* = 4), this result should be assessed with caution. In a second risk model, we were able to demonstrate that simultaneous RVI reduced CSS in M0 patients, seen for all affected fat compartments. This difference was significant for patients with PFI alone showing a 5-year CSS rate of 89% compared to 37.5% in patients with simultaneous RVI (HR 3.54, *p* = 0.043). In patients with SFI or combined PFI + SFI, each as a single variable, and for the combination with RVI, there was no statistically significant difference in terms of CSS between the respective groups (*p* = 0.409 and *p* = 0.202). However, the 5-year CSS rate was reduced in groups with simultaneous RVI (82.6% vs. 60.6% for SFI and 85.7% vs. 57.5% for PFI + SFI, see Fig. 2f, g). In summary, these two unweighted prognostic risk stratification models could confirm the different prognosis in our inhomogeneous pT3a collective. The largest difference in terms of CSS was seen between patients with combined PFI + SFI with simultaneous RVI and patients with sole PFI (5-year CSS 57.5% vs 89.0%, HR 4.01, *p* = 0.029).

## Discussion

The TNM classification has a great importance for risk stratification of RCC patients after tumor resection. Based on the 7th TNM edition from 2010, RCC with PFI, SFI or RVI alone or as a combination of these various options were summarized as pT3a tumors [4]. In the present study, we retrospectively examined the prognostic value of FI and also depending on the different fat compartments as well as RVI for medium-term CSS using 184 non-metastatic and metastatic RCC patients from an intern register. On univariate analysis for the total pT3a collective, the risk of tumor-dependent death was not significantly higher in patients with FI compared to sole RVI (HR 1.18, *p* = 0.591). These discrepancies may result from the different cohort characteristics, since exclusively pT3a patients were included in our study and only 31 of these patients showed no FI but pure RVI. Previous work on the impact of FI on RCC patient outcome showed contradictory results, ranging from a negation, as with Siemer et al. and Gilbert et al., or the dependence on tumor diameter, as in Gofrit et al., to the reliable proof of an influence, reported by Brookman-May et al. [2, 5–7]. In our study, patients with FI and simultaneous RVI showed poorer CSS compared to patients with only FI (*p* < 0.01). This is consistent with other studies that have shown poorer CSS with a 2.6-fold increased risk of cancer-related death in patients with combined FI and RVI compared to cases with either of them alone [8, 9]. At this point, the question arises whether the presence of a combined FI and RVI should be assessed differently in the TNM classification to better stratify patients with poor prognosis.

Taking into account the different fat compartments in the present work, there was no significant difference in the univariate analysis for the whole collective as well as the M0 subcollective for patients with PFI or SFI (*p* = 0.079 and 0.667). However, Thompson et al*.* described tumors with SFI as more aggressive compared to those with PFI with a 1.63-fold higher risk of tumor-dependent death [10]. In contrast, two recent studies found no influence of the location of FI on CSS in T3a RCC patients. [11, 12]. In our cohort, the survival of patients with combined PFI + SFI was significantly worse than in patients with only PFI or SFI (*p* < 0.01 and *p* = 0.023) as well with no FI but sole RVI (*p* = 0.025). In the associated multivariate analyses, combined PFI + SFI could be confirmed as an independent negative prognostic factor for CSS in comparison to FI in only one of the two compartments (HR 1.82, *p* = 0.029). However, combined PFI + SFI could not be identified as an independent negative prognostic factor for CSS compared to sole PFI (*p* = 0.172) or SFI (*p* = 0.062). In recent studies by Kume et al., Kresowik et al. and Bedke et al., a combined PFI + SFI represented an independent risk factor in multivariate analysis for a shorter CSS in RCC [13–15]. In contrast, Poon et al. and Margulis et al*.* could not confirm combined PFI + SFI compared to sole PFI as an independent predictor [11, 12]. When comparing the described studies, it should be taken into account that both, the included patients, ranging from only localized RCC to metastatic diseases, and also the tumor classification differ significantly, since most studies used the TNM classification of 2002 [5, 7, 16]. Despite the intensive and careful documentation of the available data, the retrospective study design is a limiting factor and although the primary histopathologic analysis was made by uro-pathologists, no pathology re-review of the parrafin embedded tissue and the respective slides was performed. In the two unweighted models for prognostic risk stratification, we were able to show, on the one hand, the large difference in CSS in pT3a RCC patients ranging from 5-year CSS rates of 91.4% in patients with FI and the parameters M0 L0 RVI0 to 44.9% in M0 L1 RVI1 patients (HR 4.89, *p* < 0.01). On the other hand, we could demonstrate that CSS was significantly reduced in M0 patients of each affected fat compartment when RVI was present at the same time. The largest margin in terms of CSS was found between patients with combined PFI + SFI and additional RVI and patients with PFI alone without RVI (5-year CSS 57.5% vs. 89%, HR 4.01, *p* = 0.029). This observation is consistent with the studies by Guo et al*.* and Shah et al*.*, who showed a significantly worse prognosis for patients with combined PFI + SFI and RVI [17, 18].

## Conclusions

Taking into account the data obtained, the question arises whether the current TNM classification is sufficient to predict the risk of recurrence and survival of patients with pT3a RCC, or whether a division into a combined PFI + SFI compared to invasion in only one fat compartment and in particular the consideration of an additional RVI would be an important step towards better risk stratification. Furthermore, questions arise about the therapeutic consequences for patients with pT3a RCC with combined PFI + SFI with or without RVI. Some options could include a more frequent follow-up regime and review of adjuvant therapies to improve the expected poorer survival in these patients. In addition, this retrospective study highlights the importance of a careful, standard assessment of the type of FI. It is clear that larger studies will be required to further validate the prognostic value of combined PFI + SFI with simultaneous RVI in pT3a RCC.

## Abbreviations

CI: Confidence interval
CSS: Cancer specific survival
FI: Fat invasion
G: Grading
PFI: Perirenal fat invasion
HR: Hazard ratio
L: Invasion into lymph vessels
M: Distant metastasis
N: Regional lymph nodes
R: Resection status
RCC: Renal cell carcinoma
RVI: Hilar vein involvement
SFI: Sinus fat invasion
T: Primary tumor
TNM: Tumor-node-metastasis classification system
V: Invasion into veins
